# Investigating ****γ****H2AX as a Biomarker of Radiosensitivity Using Flow Cytometry Methods

**DOI:** 10.5402/2013/704659

**Published:** 2013-04-10

**Authors:** Lindsay A. Beaton, Leonora Marro, Shawn Malone, Sara Samiee, Scott Grimes, Kyle Malone, Ruth C. Wilkins

**Affiliations:** ^1^Environmental and Radiation Health Sciences Directorate, Health Canada, 775 Brookfield Road, Postal Locator 6303B, Ottawa, ON, Canada K1A 0K9; ^2^Department of Physics, Carleton University, 1125 Colonel By Drive, Ottawa, ON, Canada; ^3^The Ottawa Hospital Cancer Centre, 501 Smyth Road, Ottawa, ON, Canada

## Abstract

*Background and Purpose*. This project examined the *in vitro* **γ**H2AX response in lymphocytes of prostate cancer patients who had a radiosensitive response after receiving radiotherapy. The goal of this project was to determine whether the **γ**H2AX response, as measured by flow cytometry, could be used as a marker of individual patient radiosensitivity. *Materials and Methods*. Patients were selected from a randomized clinical trial evaluating the optimal timing of Dose Escalated Radiation and short-course Androgen Deprivation Therapy. Of 438 patients, 3% developed Grade 3 late radiation proctitis and were considered to be radiosensitive. Blood was drawn from 10 of these patients along with 20 matched samples from patients with Grade 0 proctitis. Dose response curves up to 10 Gy along with time response curves after 2 Gy (0–24 h) were generated for each sample. The **γ**H2AX response in lymphocytes and lymphocyte subsets was analyzed by flow cytometry. *Results*. There were no significant differences between the radiosensitive and control samples for either the dose course or the time course. *Conclusions*. Although **γ**H2AX response has previously been demonstrated to be an indicator of individual patient radiosensitivity, flow cytometry lacks the sensitivity necessary to distinguish any differences between samples from control and radiosensitive patients.

## 1. Introduction

 The severity of late normal tissue toxicity is a limiting factor during the administration of radiotherapy [1]. It has become clear that interpatient variability in the incidence of late normal tissue toxicity could be partially due to individual patient sensitivity to radiation [2] and the prediction of a patient's radiosensitivity would facilitate improved patient treatment [3]. The induction and repair of chromosomal damage in irradiated lymphocytes are thought to be promising predictors of individual patient sensitivity to ionizing radiation [4]. Since DNA double-strand breaks (DSBs) are considered to be the critical lesion for DNA damage, the lack of repair, or misrepair, can have severe repercussions. As such, it is important to be able to quantitate the induction and disappearance of DSBs.

Rogakou et al. in 1999 [5] showed that H2AX (an electrophoretic isoform of the histone H2A) is phosphorylated (*γ*H2AX) at the sites of DSBs, and Sedelnikova et al. in 2003 [4] demonstrated that *γ*H2AX foci corresponded one to one with DSBs. These results mean that the number of DSBs induced can be measured by counting the number of *γ*H2AX foci [4]. Furthermore, by staining *γ*H2AX with a fluorochrome-labelled antibody, these individual foci can be visualized and enumerated by microscopy (spot counting) which is a sensitive but time-consuming method. Alternatively, flow cytometry can measure the emitted fluorescence from the *γ*H2AX foci, which is a less sensitive method, however, it is one that provides good population statistics.

 In 2004, Olive and Banáth [6] proposed that the expression of *γ*H2AX, as measured by flow cytometry, could be used as an indicator of tissue radiosensitivity. Rübe et al. in 2010 [7] studied the *γ*H2AX response in mice and found that even minor impairments in DSB repair lead to excessive DNA damage accumulation during fractionated irradiation and concluded that this may have a significant impact on normal tissue response in clinical radiotherapy. In addition to induction of repair as a potential indicator, Menegakis et al. in 2009 [8] investigated residual *γ*H2AX foci as potential indicators of clonogenic cells and found that after 24 hours, residual *γ*H2AX foci correlated with clonogenic survival.

While a study by Werbrouck et al. in 2010 [9] found that scoring of *γ*H2AX foci in isolated T-lymphocytes was not predictive for late radiotoxicity, a study by Chua et al. in 2011 [10] comparing the efficiency of DSB repair and chromosomal radiosensitivity in *ex vivo* irradiated blood lymphocytes between radiosensitive and control patients found that residual foci measured 24 h after 4 Gy were significantly higher in patients considered to be radiosensitive as compared to controls.

A study by Andrievski and Wilkins in 2009 [11] noted that lymphocyte subsets CD4, CD8, and CD19 had varied *γ*H2AX responses and proposed that investigating the *in vitro* response in lymphocyte subsets, CD19 in particular, might be proved to be a more sensitive assay.

The goal of this project was to use flow cytometry methods to analyze the induction and residual of *in vitro* *γ*H2AX response in lymphocytes and lymphocyte subsets of patient classified as radiosensitive or nonsensitive (control) to radiation in order to determine if the lymphocytes or lymphocyte subsets yielded a more sensitive indication of radiation response.

## 2. Materials and Methods

### 2.1. Patient Selection and Sample Collection

Patients for this study were selected from an ongoing phase 3 clinical trial (OTT0101, OHREB number 2001014-01H) evaluating the optimal sequencing of radiation and 6 months hormonal of therapy in cT1-cT3 prostate cancer (as described previously in Beaton et al. [12]). Briefly, based on the RTOG/EORTC Late Toxicity Scale [13], 10 patients who were identified with Grade 3 late proctitis (radiosensitive) were matched with 20 Grade 0 patients (control).  Table 1 (also published in [12]) provides a summary of the patients' clinical characteristics. These patients gave informed consent to give a venous blood sample. The study was approved by The Ottawa Hospital Research Ethics Board and Health Canada's Research Ethics Board. 

The blood samples, drawn in lithium-heparin tubes (Becton, Dickinson and Co (BD), Mississauga ON), were cultured 1 : 10 with sterile 15% complete media (86% RPMI 1640 (Invitrogen, Burlington, ON), 15% FBS (Sigma-Aldrich, Oakville, ON), and 1% 2 mM L-glutamine (Sigma-Aldrich, 100x)) and kept in a 25 cm^2^ vented culture flask (VWR, Mississauga, ON). From the culture flask, the blood in media suspension was aliquoted into 19 samples of 1 mL into 5 mL flow tubes (BD) and placed on ice. These 19 samples included 6 dose points, 1 unstained control, 3 single-colour controls, 1 negative *γ*H2AX colour control, 1 positive *γ*H2AX colour control, and 7 Full Minus One (FMO) controls. At least 1 mL of blood was left in the lithium-heparin tube on a rocker to be used for the time course experiments. Of this 1 mL, 100 *μ*L was removed and added to 900 *μ*L complete media before each of the 7 time point samples was irradiated.

### 2.2. Irradiation

The blood samples were irradiated upright, on ice, in a cabinet X-ray (X-RAD320, Precision X-ray, North Branford CT) at 250 kV, 12.5 mA, with a 2 mm Al filter and at 50 cm from the source. The dose rate was 1.7 Gy/min calibrated using a Radcal 9010 ion chamber (Radcal, Monrovia, CA) calibrated at NRC (*N*_*k*_ = 0.992 Gy/Gy_reading_ at 250 kV). The dose course samples received the following doses: 0, 2, 4, 6, 8, and 10 Gy. The unstained, single-colour, negative *γ*H2AX, and negative FMO controls remained unirradiated. The positive *γ*H2AX and positive FMO controls were exposed to 10 Gy. After irradiation, the samples were left to stand in a water bath at 37°C, tightly capped, for 1 hour. The time course samples received 2 Gy each and were placed in a 37°C incubator, loosely capped for the following incubation times: 0, 0.5, 1, 2, 4, 8, and 24 h, with irradiations being staggered such that all time course samples were processed simultaneously.

### 2.3. Fixing and Permeabilizing

After incubation, the samples were fixed and permeabilized using a modified method from Chow et al. [14]. The samples were cold (0°C) centrifuged for 8 minutes at 300 ×g. The supernatant was aspirated and the pellet was resuspended. 65 *μ*L of 10% formaldehyde, at room temperature (RT), was carefully added and mixed immediately. The samples were incubated at RT for 10 minutes. 1 mL of cold Triton-X in PBS (0.12% w/v Triton-X 100, Sigma-Aldrich; 100% PBS) was then added to each sample, which was again incubated for 30 minutes at RT. The samples were rinsed with 1 mL of cold wash buffer (96% PBS, 4% FBS), mixed, and cold centrifuged for 8 minutes at 300 ×g. The supernatant was aspirated, the pellet was resuspended, and 1 mL of cold methanol solution (70% MeOH, 30% PBS) was added dropwise while vortexing. The samples were maintained on ice, stored at −40°C overnight, and processed within 2 days.

### 2.4. Staining and Flowing Samples

From the freezer, the samples were resuspended and 1 mL of cold TBS was added and mixed. The samples were cold centrifuged for 5 minutes at 400 ×g, the supernatant was aspirated, and the pellet was resuspended. 1 mL of cold TST (96% TBS, 4% FBS, 0.1% Triton-X 100) was added to each sample and mixed. The samples were incubated on ice for 10 minutes and then cold centrifuged for 5 minutes at 400 ×g. After aspirating the supernatant and resuspending the pellet, 1 *μ*L of *γ*H2AX-FITC (Millipore, Etobicoke, ON) was added to the required samples and all of the samples were incubated on ice, in the dark, for 2 hours. Thirty minutes prior to the end of incubation, the CD antibodies were also added to the required samples (10 *μ*L CD4-PE (BD), 5 *μ*L CD8-APC (BD), and 5 *μ*L CD19-PC7 (Beckman Coulter, Mississauga, ON)). 

Following the 2-hour incubation, 1 mL cold TBS + 2% FBS solution was added to all the samples, and they were cold centrifuged for 5 minutes at 400 ×g. The supernatant was aspirated, and the pellet resuspended in 500 *μ*L of cold TBS + 2% FBS. The samples were stored in the dark on ice until they were run through the flow cytometer.

A gate was drawn around the lymphocyte population based upon characteristic populations in the forward versus side scatter acquisition plots. At least 50,000 lymphocytes were collected. The data was acquired without compensation on a FACSCalibur (BD), and compensation and analysis were performed after acquisition using the software FCS Express (De Novo Software, Los Angeles, CA).

### 2.5. Statistical Analysis

In each of the two experiments (dose course, time course), two status groups, sensitive and control populations, were compared. Each experiment investigated the two groups with respect to four endpoints (lymphocytes, CD4, CD8, and CD19). In both of these experiments a two-factor analysis of variance model with randomized complete block design (RCBD) was applied, where patients were nested within each level of status. The two factors are status group (sensitive, control) and dose (or time). The RCBD accounts for individuals blood samples tested at all dose groups or measured at each time point. The interest was investigating if there was a difference in the mean fluorescent intensity for the four endpoints between status groups, as well as if differences between status groups occurred at each dose group or time point.

Linear mixed effects models (LMMs) were applied to model the outcome of the mean fluorescent intensity, where each individual sample was considered a random block nested within status group, to control for correlation between samples in various dose groups or at various time points. When the overall *F* test from the LMM for difference between status groups or the interaction between status group and dose or time was observed (*P* < 0.05), then multiple comparison tests were applied to compare the two status groups at each dose level or time point. Bonferroni corrections were applied for pairwise comparisons in order to adjust the type I error rate to be less than 0.05. The endpoints lymphocytes, CD4, CD8, and CD19 were analyzed separately.

The assumptions for LMM were verified using the Anderson-Darling test for normality of residuals and Bartlett's test for homogeneity of variance across groups. When these assumptions were not satisfied then nonparametric statistics were used.

## 3. Results

**Figure 1:**
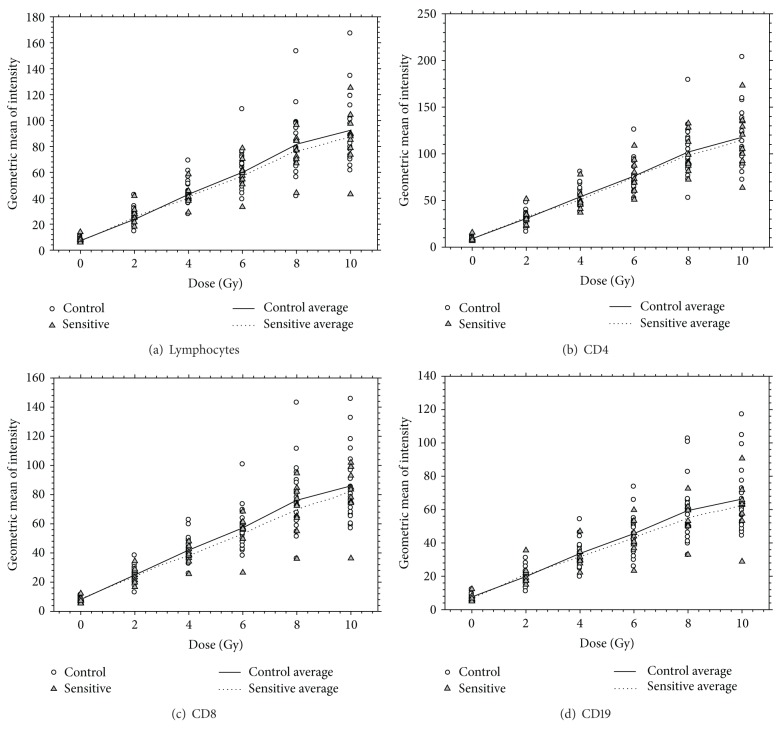
Dose course results for lymphocytes (a) and each subset ((b), (c), (d)).

Table 2 presents the average and standard deviation of geometric mean fluorescent intensity for CD4, CD8, CD19, and lymphocytes at each dose and status group in the dose course.  Figure 1 displays the results from the four endpoints CD4, CD8, CD19, and lymphocytes in the dose course. As can be seen in Table 2 and in Figure 1, average geometric mean fluorescent intensity was similar between the two status groups (sensitive and control) for each of the four endpoints. There were no significant differences observed between the two status groups at each of the respective dose groups (*P* > 0.05 in all endpoints, see Table 2).

**Figure 2:**
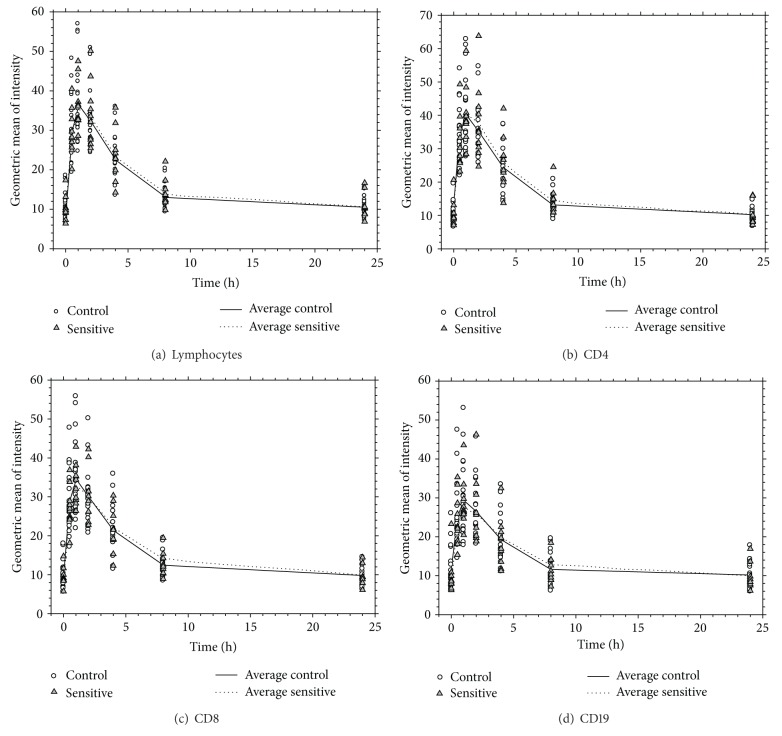
Time course results for lymphocytes (a) and each subset ((b), (c), (d)).

Table 3 presents the average and standard deviation of geometric mean fluorescent intensity for CD4, CD8, CD19, and lymphocytes at each time point and status group in the time course.  Figure 2 displays the results from the four endpoints CD4, CD8, CD19, and lymphocytes in the time course. As can be seen in Table 3 and in Figure 2 average geometric mean fluorescent intensity was similar between the two status groups (sensitive and control) for each of the four endpoints. As well there were no significant differences observed between the two status groups at each of the respective time points (*P* > 0.05 in all endpoints, see Table 3).

## 4. Discussion

Unfortunately, after fairly rigorous examination of the patient samples, with 6 dose points and 7 time points each, there was no significant difference found in either experiment.

Although it is not uncommon to normalize the data to the 0 Gy or 0 h time point, it was decided that this could potentially skew the data as those points are just as susceptible to experimental error. It has been shown (see Lew 2007 [15] for a detailed example) that when there is a correlation between the data, as is the case in this study, the randomized block ANOVA is more powerful than a one-way ANOVA. Despite this, no significant differences were found. 

In order to explore the data further, the presence of possible outliers was investigated. Absolute values of studentized residuals were generated for each patient at each dose and time point, and any greater than 2 were considered to be suspect. With some of the points identified as suspect, the statistical analysis was run on the ranks of the data and compared to the original data. By analyzing the ranks, the effect of any large or very small values on the results was removed. The results were the same in each case, indicating that the possible outliers were not masking any differences between the two groups of patients.

Although Menegakis et al. [8] found a correlation between residual *γ*H2AX foci and clonogenic survival and Chua et al. [10] found a difference 24 h after a 4 Gy dose, both performed their analyses by spot counting. While the results of this study were unable to detect any differences between the sensitive and control groups, this may have been a result of the methodology not being sensitive enough. Even with increased counting statistics, the small differences between the groups could not be detected. Higher patient numbers would help increase the sensitivity in detecting differences if they exist. It is also possible that the time and dose points used in this study were not ideal for detecting differences. Chua detected a significant difference 24 h after 4 Gy while our 24 h point was after 2 Gy. It has previously been seen that differences in radiosensitivity become more apparent when larger amounts of damage have been induced *in vitro* (Beaton et al. [12]). As the endpoints used in this study were not the same as those used by Chua et al., it is difficult to make a direct comparison. 

In conclusion, the sensitivity of conventional flow cytometry was insufficient to detect a difference between patients identified as sensitive or control, even when investigating lymphocyte subsets. Further studies will investigate whether an imaging flow cytometer, with increased sensitivity due to imaging capabilities, will be capable of distinguishing between the two populations. This technology will combine the sensitivity of spot counting with the advantage of high throughput and better count statistics.

## Figures and Tables

**Table 1:** Clinical features of control and radiosensitive groups.

Clinical group	Age at diagnosis (mean and range)	Preexisting cardiovascular disease/hypertension	Preexisting type II diabetes	Smoker/exsmoker	Mean follow-up time posttreatment (years)	Mean time to onset of Grade 3 proctitis (range)
Control	69 (60–77)	8/20	3/20	8/20	6	N/A
Radiosensitive	73 (68–76)	6/10	1/10	4/10	7	21 months (9–41 months)

**Table 2:** Comparing control to sensitive groups for the dose course experiments. Cells in table represent the geometric mean (and standard deviation) of the fluorescent intensity of the *γ*H2AX signal for each status and dose combination.

Treatment group	Dose (Gy)					
	0	2	4	6	8	10
	CD4^a^					

Control (*n* = 20)	8.83 (1.31)	30.73 (6.93)	53.91 (10.44)	75.92 (17.80)	101.98 (26.90)	117.81 (31.73)
Sensitive (*n* = 10)	8.83 (2.58)	32.53 (7.97)	51.37 (11.41)	75.33 (17.44)	98.44 (19.83)	114.41 (30.85)

	CD8^b^					

Control (*n* = 20)	8.25 (1.50)	24.82 (5.86)	42.00 (8.89)	57.09 (14.41)	76.31 (24.05)	86.12 (24.94)
Sensitive (*n* = 10)	7.84 (1.84)	25.22 (5.16)	38.79 (6.13)	53.57 (11.21)	70.45 (16.58)	82.55 (19.24)

	CD19^c^					

Control (*n* = 20)	7.61 (1.59)	19.99 (4.81)	33.81 (7.90)	45.66 (11.69)	59.37 (18.40)	66.38 (20.61)
Sensitive (*n* = 10)	6.97 (2.15)	21.07 (5.73)	31.82 (6.39)	42.91 (9.79)	55.31 (10.43)	62.32 (15.43)

	Lymphocytes^d^					

Control (*n* = 20)	8.39 (1.29)	26.49 (6.17)	45.08 (9.49)	61.32 (15.44)	81.72 (24.08)	92.39 (26.90)
Sensitive (*n* = 10)	8.32 (2.20)	27.61 (6.32)	42.10 (7.44)	58.83 (11.93)	75.97 (14.23)	87.49 (21.26)

Overall status effect: ^a^ *F*_(1,28)_ (*P* value) = 0.06 (0.81), ^b^ *F*_(1,28)_ (*P* value) = 0.38 (0.54), ^c^ *F*_(1,28)_ (*P* value) = 0.33 (0.57), ), and ^d^ *F*_(1,28)_ (*P* value) = 0.29 (0.59).

Overall difference between status groups at various dose groups: ^a^ *F*_(5,140)_ (*P* value) = 0.18 (0.97), ^b^ *F*_(5,140)_ (*P* value) = 0.33 (0.89), ^c^ *F*_(5,140)_ (*P* value) = 0.42 (0.84), and ^d^ *F*_(5,140)_ (*P* value) = 0.42 (0.84).

**Table 3:** Comparing control to sensitive groups for time course experiments after 2 Gy. Cells in table represent the geometric mean (and standard deviation) of the fluorescent intensity of the *γ*H2AX signal for each status and time combination.

Treatment group	Time (hrs)						
	0	0.5	1	2	4	8	24
	CD4^a^						

Control (*n* = 20)	11.01 (3.50)	33.38 (8.43)	40.40 (11.07)	35.40 (8.10)	24.42 (6.33)	13.23 (2.93)	10.30 (2.03)
Sensitive (*n* = 10)	10.80 (3.86)	32.18 (7.81)	40.41 (8.39)	37.91 (11.26)	25.87 (7.81)	14.25 (4.07)	10.26 (3.14)

	CD8^b^						

Control (*n* = 20)	10.09 (3.32)	29.02 (7.79)	34.44 (9.66)	29.87 (7.26)	21.31 (5.94)	12.36 (3.02)	9.72 (2.17)
Sensitive (*n* = 10)	9.13 (2.64)	27.27 (5.22)	33.18 (5.03)	30.29 (6.51)	21.26 (5.65)	12.70 (3.03)	9.37 (2.51)

	CD19^c^						

Control (*n* = 20)	11.38 (5.29)	25.14 (8.47)	29.45 (9.35)	26.54 (7.42)	19.30 (6.22)	11.64 (3.86)	10.26 (2.98)
Sensitive (*n* = 10)	9.85 (4.90)	24.18 (6.56)	28.48 (6.24)	26.25 (8.69)	18.54 (6.25)	11.56 (3.32)	9.69 (3.21)

	Lymphocytes^d^						

Control (*n* = 20)	10.54 (3.13)	30.50 (7.50)	36.92 (9.79)	32.54 (7.41)	22.82 (5.77)	13.22 (3.02)	10.72 (2.20)
Sensitive (*n* = 10)	10.28 (3.07)	29.27 (5.80)	36.59 (5.84)	33.99 (7.99)	23.65 (6.37)	14.03 (3.47)	10.72 (3.05)

Overall status effect: ^a^ *F*_(1,28)_ (*P* value) = 0.05 (0.82),^b^ *F*_(1,28)_ (*P* value) = 0.11 (0.74), ^c^ *F*_(1,28)_ (*P* value) = 0.15 (0.70), and ^d^ *F*_(1,28)_ (*P* value) = 0.01 (0.94).

Overall difference between status groups at the various time points: ^a^ *F*_(6,167)_ (*P* value) = 0.38 (0.89), ^b^ *F*_(6,167)_ (*P* value) = 0.25 (0.96),^c^ *F*_(6,167)_ (*P* value) = 0.08 (1.00), and ^d^ *F*_(6,167)_ (*P* value) = 0.27 (0.95).
